# Notes of the Week

**Published:** 1921-08-06

**Authors:** 


### NOTES OF THE WEEK.

### IDEAS FOR HOSPITAL WEEK.

If opinions are divided upon the possibility of a successful hospital pageant, the hospital fair or week's carnival has never been more fashionable. In London, not only has there been the London Hospital carnival week at Stamford Bridge, which ended on July 30, but a similar week was held at Catford for the benefit of the Seamen's, the Miller, St. John's (Lewisham), the Blackheath, and the South-Eastern Children's Hospitals, and the Sydenham Infant Welfare Centre. The object of the fair was to raise money for the Hospital Welfare Society, which supports the above-mentioned institutions. In an interesting speech, Mr. W. T. Allen, the Society's chairman, said that the weekly collection was the main thing. In January 1920 this was 27s. 8d. In one week during the past July it was £120. The three boroughs were divided into ten sections, and they hoped to raise £300 a week, or £15,000 a year. That shows what can be done in London by weekly collections, and the two carnivals at Stamford Bridge and at Catford provide useful experience for the new committee now considering how to organise a Hospital Week in the spring. With the programmes and organisation of these two efforts before it, the new committee should have something to go upon, and if it can enlist the interest of the organisers of these two carnivals so much the better. The new committee could hardly have been formed at a more opportune time.

### THE POOR LAW AND THE VOLUNTARY HOSPITALS COMMISSION.

The personnel of the Voluntary Hospitals Commission has been criticised because it contains no representative of the Poor Law, in spite of the fact that the Commission is expected to raise the questions of the use of vacant beds in infirmaries and the possibility of their co-operation with the voluntary hospitals. Since the prime business of the Commission is the distribution of the Government grant, it is easy to make a technical defence of this omission, but it is because the work of the Commission is not limited to the distribution of the grant that the criticism has been made. Since, too, the grant is expected to go to those institutions most in need, and to those which convince the Commission that they have used every means to improve their position, it is possible that attempts at co-ordination with infirmaries may be regarded as a qualification. In short, it is not easy to see exactly where, when, and how far the Commission will be concerned with the Poor Law, and this uncertainty has made the latter apprehensive. In our view, until there is any statutory change in the position and powers of the guardians in respect to the use to which the infirmaries may be put, there is not much substance in this criticism.

### THE FIRST-AID SERVICES.

Many of our readers will appreciate the claim of Sir James Cantlie that first-aid and ambulance work should be regarded as a branch of special surgery. There is certainly some cause for protest against the conditions and circumstances in which this subject is still of necessity taught. A matter which becomes, in this mechanical age, daily of greater importance is certainly deserving of treatment upon correct collegiate and institutional lines. Sir James gave his audience at Newcastle many graphic illustrations, from the farms, the mines, and the cities, of the urgent need for more widespread and thorough "first-aid" teaching, and his statement that there is no reason why every man should not have a knowledge of ambulance, and no reason why all cities and industrial centres should not have their college of ambulance, applies to the whole country, quite apart from the particular needs of Newcastle.

### CANCER RESEARCH.

Some of the comments made at the annual meeting of the Imperial Cancer Research Fund might well have been used by the press to illustrate in what direction cancer research really moves. As the Duke of Bedford said, in the investigation of any disease the discovery of a sure method of producing that disease in previously healthy individuals is a landmark of the first importance. In the case of cancer this, in animals, has been achieved during the year. The work of Japanese and Danish investigators, producing cancer in mice by applications of tar and coal-tar derivatives, has been considerably extended in this country. From this beginning an analysis of the conditions under which cancer arises is being continued with every prospect of gradual success. In short, it is the cause and not the universal "cure" that is claiming this particular attention.

### FOR SERVICE TO HUMANITY.

The late Dr. R. Murray Leslie, the donor of the Gold Medal of the Royal Society of Medicine, of which the first award has been made to Sir Almroth Wright, desired that it should be awarded to the scientist, man or woman, who made the most valuable contribution to the science and art of medicine. The first recipient was to be one whose work had been of the greatest use to military medicine and surgery during the late war. In these circumstances the claims of Sir Almroth Wright are indisputable, and it is also satisfactory to know that, though the founder of the award is deceased, he lived long enough to know who the recipient was to be, and had heartily approved of the choice.

### THE "JAPAN MEDICAL WORLD."

Our friends the Japanese are progressive in most of life's activities, and not least in things pertaining to medicine and the allied sciences, but we have long regretted the inaccessibility of early news by reason of the language difficulty. The difficulty is now overcome, and the first issue of the *Japan Medical World*, No. 1, Tokyo, May 1921, has been published. The style and form of the journal are excellent, and this first issue contains an interesting *résumé* of medical progress in Japan during the past fifty years. Attention is called to the fact that there are twenty-two medical colleges in Japan, all well equipped, as well as specially endowed institutions, such as the Kitasito Institute for Infectious Diseases. We welcome the appearance of this journal as a medium by which Japanese medical activities can be introduced to the English-speaking world. It is to be published monthly in English; the published price is six dollars per annum, and the offices are at 10 Omote-Jimbo-cho-Kanda, Tokyo.

### THE "JOURNAL OF OBSTETRICS AND GYNÆCOLOGY."

The publication of the *Journal of Obstetrics and Gynæcology of the British Empire* was suspended during the war; but the enterprise, we are glad to see, has not been allowed to expire, and republication has recently started with a new series. The editors of the *Journal* are Drs. Comyns Berkeley and Eardley Holland, whose initial issue shows that they intend to maintain and extend the high reputation earned by their predecessors. The most important individual contribution in a very interesting list is that of Mr. Gordon Ley on the subject of what is usually called accidental hæmorrhage, for which he prefers the term utero-placental hæmorrhage. He claims to have established the fact that this complication of pregnancy is always due to a toxæmia, which can be diagnosed beforehand, because it causes albuminuria and other symptoms. If this claim, the case for which is very ably argued, is sustained on impartial investigation, the bearings of Mr. Ley's work on treatment, and especially on prophylactic treatment, are obvious: incidentally, it may be mentioned that a paper later on in the *Journal*, by Dr. Fletcher Shaw, supports Mr. Ley's view, and shows also how its adoption will influence current teaching about treatment. Lastly, the format of the new series is an improvement on that of pre-war days: but there are too many misprints, and the selection of types for differentiating purposes betrays inexperience (*e.g.*, pp. 145 and 151). We wish the new venture every success. The price is 12s. per quarterly number, or two guineas per annum.

### ADMISSION OF ACCIDENT CASES.

As a result of the action of the Lambeth Board of Guardians in directing the attention of the responsible authorities to cases of serious injury arising out of street accidents being first taken to general hospitals and then transferred to Lambeth Infirmary, a conference has been held, at which representatives of the Ministry of Health and the London County Council, and Mr. Frank Briant, M.P., Chairman of the Lambeth Board of Guardians, with their medical officer at the infirmary and the clerk were present. There was a full interchange of opinion, and, while no decision was come to on the matter, it was understood that in the event of the occurrence of any similar cases in future, where the successful treatment of a patient is jeopardised by delay in admission, further representations are to be made jointly to the responsible quarters.

### AN EXAMPLE TO BIRMINGHAM.

The board of management of the General Hospital, Birmingham, has decided to close from August 1 two wards containing fifty beds, which will reduce the number to three hundred and fifty. The waiting-list at present numbers five hundred, and of course the decision is a serious one, because it follows the recent decision to close thirty beds at the Jaffray Branch Hospital. The committee is also understood to suggest the co-ordination of appeals especially to employers and wage-earners. Perhaps the joint action of the London Hospital, St. Thomas's, and the Royal Free in adopting the Sussex scheme out of the three alternatives suggested by King Edward's Hospital Fund Policy Committee may encourage similar action in Birmingham. Everything shows that one " practical experiment " is more stimulating than much discussion, and if co-operation is possible in London, where independence has been the rule hitherto, it should be equally possible in Birmingham.

### THE SHEFFIELD CONTRIBUTION SCHEME.

The Advisory Committee of the Sheffield Printing Trades, of which Mr. W. H. Hartley is President, has been considering the claims of the Sheffield Joint Hospitals Contribution Fund. Hitherto some 350 trade unions have had the scheme submitted to them, and 120 firms have agreed to it. The help of the Federated Trades' Council has been specially valuable, and now that the Advisory Council of the Printing Trades has recommended its constituents to adopt the scheme the number of these should be increased. The representatives wished to be satisfied that subscribers would be distinguished from non-contributors (presumably by being relieved of any payment when in hospital); and that, it is remarkable to note, the complaints occasionally made of inconsiderate treatment were gone into. A point has also been made that these weekly contributions involve a large amount of extra bookkeeping, which, though voluntarily carried out, is onerous. The answer seems to be that this difficulty has nowhere prevented a contribution from being started or carried on where there was the wish to do either, and that to name the bookkeeping as a difficulty is to manufacture obstacles.

### SPIRIT DUTY VOLUNTARY HOSPITALS) GRANT.

We are officially informed that the Ministry of Health and the Scottish Board of Health are prepared to receive applications from voluntary hospitals for grants in respect of payment of duty involved by the use of duty-paid spirit or drugs containing duty-paid spirit for medical and surgical purposes in these hospitals during the year 1920. Forms of application have already been sent to those hospitals to which a grant was paid last year. Any other hospital which desires to make application should communicate immediately with the Secretary to the Ministry of Health, Whitehall, or the Secretary to the Scottish Board of Health, Edinburgh, as the case may be.

